# Measuring Family Members' Satisfaction with End-of-Life Care in Long-Term Care: Adaptation of the CANHELP Lite Questionnaire

**DOI:** 10.1155/2017/4621592

**Published:** 2017-06-19

**Authors:** Shevaun Nadin, Mohammed Ali Miandad, Mary Lou Kelley, Jill Marcella, Daren K. Heyland

**Affiliations:** ^1^Centre for Education and Research on Aging and Health and Department of Health Sciences, Lakehead University, 955 Oliver Rd., Thunder Bay, ON, Canada P7B 5E1; ^2^School of Social Work and Centre for Education and Research on Aging and Health, Lakehead University, 955 Oliver Rd., Thunder Bay, ON, Canada P7B 5E1; ^3^North West LHIN Regional Palliative Care Program with St. Joseph's Care Group, 35 N. Algoma St., Thunder Bay, ON, Canada P7B 5G7; ^4^Department of Critical Care Medicine, Queen's University, Watkins 5, Kingston General Hospital, Kingston, ON, Canada K7L 2V7

## Abstract

**Rationale:**

Improving end-of-life care (EOLC) in long-term care (LTC) homes requires quality measurement tools that assess families' satisfaction with care. This research adapted and pilot-tested an EOLC satisfaction measure (Canadian Health Care Evaluation Project (CANHELP) Lite Questionnaire) for use in LTC to measure families' perceptions of the EOLC experience and to be self-administered.

**Methods and Results:**

* Phase 1*. A literature review identified key domains of satisfaction with EOLC in LTC, and original survey items were assessed for inclusiveness and relevance. Items were modified, and one item was added.

**Phase 2:**

The revised questionnaire was administered to 118 LTC family members and cognitive interviews were conducted. Further modifications were made including reformatting to be self-administered.

**Phase 3:**

The new instrument was pilot-tested with 134 family members. Importance ratings indicated good content and face validity. Cronbach's alpha coefficients (range: .88–.94) indicated internal consistency.

**Conclusion:**

This research adapted and pilot-tested the CANHELP for use in LTC. This paper introduces the new, valid, internally consistent, self-administered tool (CANHELP Lite Family Caregiver LTC) that can be used to measure families' perceptions of and satisfaction with EOLC. Future research should further validate the instrument and test its usefulness for quality improvement and care planning.

## 1. Introduction

Every person deserves to receive quality care at the end of life, and developing quality end-of-life care (EOLC) across diverse settings is an international priority [1–3]. An essential part of providing quality EOLC is creation of quality measurement tools, including ones that measure families' satisfaction as an important indicator of quality care [4–8]. This paper introduces a new instrument for measuring families' perceptions of quality of EOLC in LTC. This instrument makes an important contribution in three ways. It measures both the importance of quality care to families of LTC residents and their satisfaction with EOLC, allowing the two to be compared to identify disparities. It measures satisfaction during the care experience as opposed to following the resident's death (bereavement). It is also formatted to be self-administered and can be mailed out to families, making it practical for use in the LTC setting. LTC homes often lack human resources to conduct face-to-face interviews for quality improvement.

Long-term care has become a major location of death in Canada. The average age of Canadian LTC residents in 2015-2016 was 83 years. Their most common health conditions were heart/circulation diseases (70.8%), neurological diseases including dementia (78.5%), endocrine/metabolic/nutritional diseases (39.9), and psychiatric/mood diseases (37.6) [9]. Many residents had multiple diagnoses and over 90% died of chronic disease as opposed to cancer [9]. Given advanced age and comorbidities, it is not surprising that almost 20% of residents die each year, with most of them dying in their LTC home rather than being transferred to hospital or hospice [9–11]. There is now a normalcy to caring for people who are dying in LTC homes [12, 13]. This trend exists throughout the developed world and many countries have initiated efforts to improve EOLC in LTC homes to better meet the needs of their residents and families [14–17].

The philosophy of palliative care (PC) in Canada has now shifted to include the needs of an aging population who are dying of chronic diseases such as heart disease, dementia, or frailty, thus encompassing LTC residents [18, 19]. PC is no longer viewed as specialized care offered to cancer patients who are imminently dying in a hospital or a specialized hospice setting. For those chronically ill, older people, PC is now seen as an approach to care aimed at preventing and relieving suffering and enhancing quality of life both for them and their families [20, 21]. The palliative approach advocates caring for residents in LTC until the end of their lives [22].

The palliative approach promotes early identification and is best understood as an added layer of support which benefits residents in the last year of life [23, 24]. The focus is symptom management, with care plans evolving based on need rather than diagnosis or prognosis [24]. EOLC is the final phase of the palliative approach, initiated when residents are seriously ill, are deteriorating, and are expected to die soon. Thus, providing quality EOLC is an essential component of providing the palliative approach in LTC.

The research presented here was conducted in 2013 as part of the Quality Palliative Care in Long-Term Care (QPC-LTC) Alliance research. QPC-LTC was a five-year (2008–2013) participatory action research project which aimed to improve the quality of life of people dying in LTC homes by formalizing PC programs within each home. Working with four LTC homes in Ontario and using a comparative case study design, innovations to implement the palliative approach to care were undertaken on each site. Using findings from comprehensive organizational assessments, the researchers developed a new framework and over 40 policy, practice, and educational resources to support residents, families, and staff when implementing the palliative approach [10, 12, 25–36]. These QPC-LTC resources were assembled into a toolkit that is housed on an open-access website [25]. However, researchers identified an unmet need for a measurement tool that could be used routinely by managers in LTC and guide quality improvement efforts. To address this gap, researchers undertook this research to develop an appropriate measure for family caregiver satisfaction with EOLC.

The choice to focus on families' (as opposed to residents) perceptions of care was made for three important reasons. First, while assessing residents' perceptions is acknowledged to be very important, collecting data from residents is challenging from an ethical and practical perspective. Over half are older than 85 years, over 60% have dementia, and 57% have health instability [9]. All residents have a legally appointed Power of Attorney for Personal Care to make their health care decisions when they are not deemed competent.  Table 1 summarizes the characteristics of Ontario LTC home residents, including those LTC homes in this research.

Second, best practices in PC and EOLC identify the unit of care as patient and family [20, 21]. In LTC, understanding family experience and satisfaction at end of life is even more important due to the need for family members to make health care decisions on residents' behalf. Families are thus considered to be recipients of care, having their own perspectives about care delivery and needing support and information [37].

Third, having a self-administered mail-out survey was deemed important for practical reasons, since LTC homes do not normally have staff available to administer surveys or conduct quality improvement interviews. Very few residents are capable of independently completing a survey such as CANHELP due to health status, functional status, and dementia. However, in most cases, there is a family member who is capable and willing to complete a mail-out survey.

The researchers reviewed available family satisfaction measures for palliative and end-of-life care [4–7]. Although many studies have measured quality of life in LTC settings [38–44], fewer measured quality of care provided. Most studies that did measure quality of care used nonvalidated assessment tools [4]. Most studies examined EOLC from the bereaved family's perspective (i.e., survey completed after resident's death) as opposed to measuring the family's perspective of care during the episode of care [45–53].

In the absence of a validated Canadian tool to measure families' perceptions of quality of care in LTC during the episode of care, this research focused on adapting the Canadian Health Care Evaluation Project (CANHELP Lite) Caregiver Questionnaire [54, 55] to assess EOLC in LTC. The CANHELP instrument has been previously tested for validity in a multicentre, cross-sectional study involving patients with advanced, life-limiting illnesses and their family caregivers in a hospital setting [54, 56]. While several versions of the CANHELP Questionnaire are available [57], this research focused on the CANHELP Lite Individualized Caregiver Questionnaire which was shortened by the original authors from 40 to 21 items to make it more feasible for clinical, research, and administrative purposes [55]. The QPC-LTC chose to adapt the CANHELP to leverage a credible instrument and assess how well it can measure satisfaction with EOLC in LTC setting.

CANHELP Lite Individualized Family Caregiver Questionnaire (from here on referred to as CANHELP) is a validated Canadian instrument to measure quality of palliative and EOL care [36]. This researcher-administered tool is comprised of 21 items, each indicating an aspect of quality EOLC [55]. The unique feature of this instrument in comparison to other tools is that it includes two ratings scales (importance and satisfaction scales). The importance scale rates various aspects of care delivery known to be important in EOLC from families' perspectives. The satisfaction scale allows researchers to capture family satisfaction with the level of care actually provided. Perceived importance and satisfaction can be compared for each aspect of care to identify potential opportunities for improvement in care delivery at either the patient level or organizational level [56]. By understanding families' level of satisfaction with key elements of EOLC, LTC homes will be better able to improve residents' individual care and to introduce quality improvement initiatives to the LTC sector.

The CANHELP instruments have a similar structure. Using five-point Likert scales, respondents rate each item twice: once to indicate the importance of each item to them (1 = not at all important; 5 = extremely important) and once to indicate their level of satisfaction (1 = not at all satisfied; 5 = completely satisfied). The satisfaction ratings can be averaged to obtain an overall satisfaction score as well as five domain scores (communication and decision-making, illness management, characteristics of doctors and nurses, your involvement, and relationship with doctors) [54, 55]. Juxtaposing the satisfaction ratings next to the importance ratings can be used to identify goals for improvement in care [56].

In summary, the CANHELP was chosen for this research because it measures both importance and satisfaction while receiving care and because it is a brief, validated measure with applicability across diverse EOLC contexts [54, 55]. However, the CANHELP was developed in hospital settings and had not yet been leveraged in the LTC environment. Adaptation was undertaken because the organizational context, residents' disease profiles and trajectories of dying, and staffing patterns in LTC differ considerably from other contexts [45, 58, 59], and the tool could not be applied to LTC without revision. Thus, this research sought to adapt the CANHELP for applicability to LTC. The main objective was to create a self-administered version of the CANHELP which could be used to measure families' perceptions of EOLC in LTC.

## 2. Methods

Adaptation of the CANHELP was a three-phase process. First, the original survey items were assessed for relevance to LTC by the researchers and LTC experts in the QPC-LTC Alliance, and modifications were made. Next, the questionnaire was administered to a sample of family members and, through an iterative process called cognitive interviewing, further modifications were made. Third, the properties of the final instrument were evaluated. The study was approved by the Lakehead University research ethics board (REB) and the REBs of the participating LTC sites. All participants provided consent to participate in the research. The three phases of the study method are described below.

### 2.1. Phase 1: Evaluation and Adaptation of the Questionnaire Items for Relevance in LTC

This phase evaluated the applicability of the original instrument to LTC by assessing whether any items needed to be added, removed, or modified. The instrument terminology was assessed to determine if any wording or content modifications were required to reflect the LTC context. The item content was also assessed to determine whether the questionnaire tapped into domains of EOLC relevant to LTC. To aid in this step, an extensive literature review was conducted to identify the factors that influence family members' satisfaction with EOLC in LTC. (The results of the literature review are presented below in the findings section.) These factors were compared to the survey items to determine whether any items needed to be added (i.e., factors emerging from the literature were not represented in the original tool) or removed (i.e., questionnaire items did not reflect factors that emerged in the review) [60].

### 2.2. Phase 2: Further Adaptation of the Questionnaire Based on Cognitive Interviewing

This phase used the cognitive interviewing technique to determine if any further revisions to the instrument were required. Participants were family members of LTC residents recruited from two LTC homes that participated in the larger QPC-LTC project. Invitations to participate were sent to the family member who was the Substitute Decision Maker or Power of Attorney for Personal Care, for each resident (*N* = 344), and follow-up phone calls were made to schedule interviews with interested families. The Power of Attorney for Personal Care, in Ontario, is a person designated in a legal document to make personal care decisions on behalf of a mentally incapable person [61]. All Powers of Attorney for Personal Care were family members who visited the LTC home regularly. In other jurisdictions, the Substitute Decision Maker may be called health care proxy.

During the interviews, the instrument created from Phase 1 was administered by a trained graduate student. The questionnaire was followed by a cognitive interview—a technique commonly used to obtain thorough feedback on questionnaires when they are being developed or refined [62]. The cognitive interviews sought to understand the four main cognitive processes respondents use to answer the questionnaire and to assess their perceptions of understandability and relevance of the instrument.  Table 2 illustrates the interview questions and their rationale [63]. All cognitive interviews were audio-recorded for quality purposes to ensure that responses were accurately captured and incorporated in the revision process.

Although the diagnostic profile of the residents in the participating LTC homes was consistent with the Ontario LTC resident data presented in Table 1, the researchers wanted a descriptor of each resident's individual status to examine if status influenced caregivers' responses. Therefore, a tool called the Palliative Performance Scale (PPS) [64] was introduced. PPS is a Canadian instrument used to measure performance status of palliative patients across settings of care [65, 66]. A senior nursing student experienced with using the PPS assessed each resident at the time of the interview to allow the researchers to have a consistent measure of residents' status using a measure commonly used in PC.

PPS scores can range from 100% to 0%, with decreasing scores indicating decreased status and consequent need for increased care and support (e.g., a score of 100% indicates full ambulation, requiring no support; a score of 30% indicates totally bed bound needing total care; and 0% indicates death) [64]. A PPS score is determined by ambulation, activity level and evidence of disease, self-care, intake, and conscious level [64]. The final score is heavily weighted to the domains of ambulation, activity level, and self-care. Another Canadian research has shown that reduced level of resident activity was a strong predictor of imminent death (within 31 days) in LTC homes in Ontario—a stronger predictor than demographics, diagnosis, and other health conditions [10]. The PPS is descriptive (not diagnostic) and is an effective tool for quickly describing the resident's current functional level [67]. This makes it practical for use by LTC staff, many of whom are nonregulated caregivers (not registered nurses), and it is being adopted by many Canadian LTC homes.

Revisions to the CANHELP questionnaire were made iteratively as suggested by the cognitive interviews. The cognitive interviewing continued until no further revisions were required. The final questions were reformatted as a self-administered instrument that could be mailed out to families.

### 2.3. Phase 3: Evaluation of Response Tendencies and Internal Consistency of the Adapted Questionnaire

In this phase, the revised self-administered instrument was pilot-tested and the instrument properties were evaluated. Participants were family members who were recruited from two different LTC homes. The product from Phase 2 was mailed to the Substitute Decision Maker or Power of Attorney for Personal Care for each resident of the two LTC homes (*N* = 178). If the Power of Attorney had not visited the resident in the last month, the instruction was for the survey to be completed by another family member who had visited regularly. This ensured that the family member respondent was well informed.

Descriptive statistics were examined to assess the proportions of respondents rating each response option and evaluate potential ceiling and floor effects. Attention was paid to the importance frequencies as an indication of content validity. High importance ratings would validate the items as elements of care that are important to family members. Attention was also paid to the frequencies of a newly added “do not know/no basis to judge” response option. High proportions of respondents (>10%) selecting this option on many of the items could indicate random or thoughtless responding and suggest that this is not an appropriate response category. However, a limited proportion of endorsements would validate the addition of the category, suggesting that it is a genuine response in the context of LTC.

Cronbach's alpha coefficients were examined to evaluate the internal consistency of the scales. Coefficients ≥ .80 were considered good [68].

## 3. Results

### 3.1. Phase 1

The literature review conducted at the onset of the research revealed seven domains that influence family satisfaction with EOLC in LTC.

*Communication and Interaction with Staff*. Communication and interpersonal relationships with LTC staff are important factors that shape family satisfaction [45, 50, 51, 69–71]. Families desire frequent, honest, open, and up-to-date communication about the resident's status as well as the care being provided [45–47, 58]. They also want to be counseled about resident's prognosis, have discussions with staff about EOL and comfort care measures, and be able to express concerns and fears to them [69, 72]. Compassionate, empathic, and supportive behavior from LTC staff toward the resident and family members also shapes satisfaction [47, 69, 73–76].

*Pain and Symptom Management. *Pain is a common, often underreported problem in LTC homes which is concerning to families [45, 49, 77–83]. Families have lower satisfaction when they perceive that their loved one's pain is not being adequately managed [82].

*Physician Presence and Contact. *Physician presence and contact is often low in LTC which can frustrate family members [51, 79, 83, 84]. Family members can perceive this lack of physician engagement to mean staff lack understanding of the resident's medical history, complicating EOL decision-making [46].

*Psychosocial, Spiritual, and Bereavement Support. *Providing opportunities for families to discuss residents' care needs and offering emotional support for bereaved families are important parts of satisfaction with EOLC in LTC [73, 85–87].

*Hospital Transfers and Location of Death. *Hospital transfers from LTC are frequent occurrences at end of life [88]; however, families are more satisfied when their relative dies in the LTC facility (versus hospital) [45]. This finding is similar to community research that finds that families are less satisfied with care when their loved ones do not die at home [89, 90]. Thus, LTC facilities should seek to avoid hospital transfers near end of life and proactively discuss preference for location of death with families and residents.

*Advance Care Planning. *Families want to have discussions about the resident's expressed wishes with staff, and having an advance care plan that guides the decision-making of the Power of Attorney for Personal Care significantly increases family satisfaction with end-of-life care in LTC [68, 91–93].

*Staffing Levels and Staff Education. *There is a positive relationship between staffing levels and satisfaction with care in LTC [94–98]. The importance of LTC staff receiving EOLC education has also been noted [45, 47, 97, 98].

#### 3.1.1. Revisions to the Questionnaire

**Figure 1:**
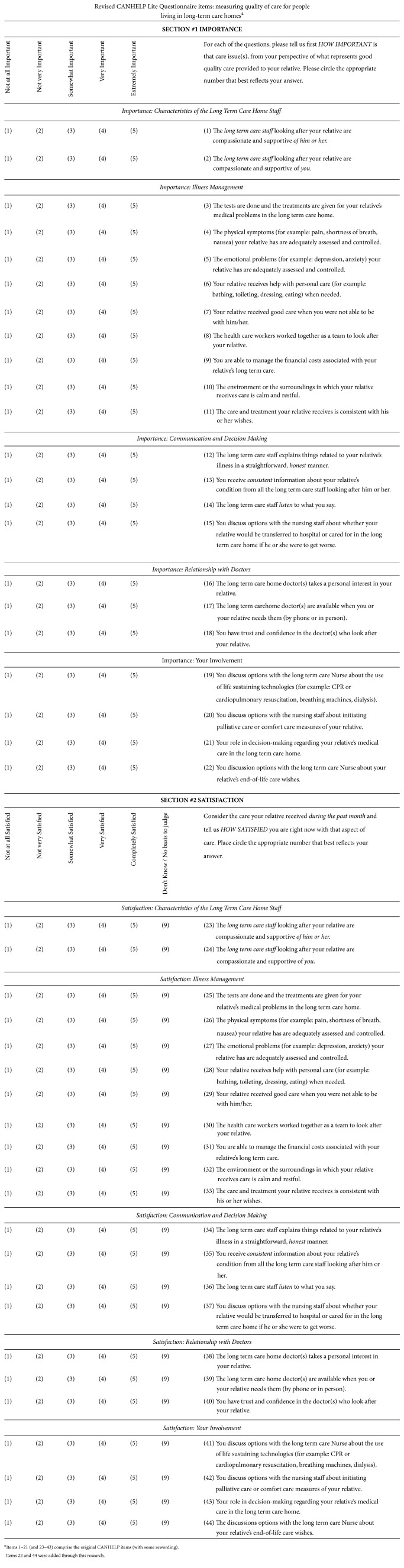


It was determined that most of the factors relevant to LTC were covered by the original survey, but an additional item that probes having EOL discussions should be added. Thus, the item “you discuss options with the nursing staff about initiating palliative care or comfort care measures of your relative” was added (item (20) in Figure 1).

No items were deemed irrelevant to the LTC context; thus, none were removed. However, terminology changes were made to some items and subscales to make them more reflective of the differences in presence of physicians, care delivery model, and staffing in LTC (namely, the terms “nurses” and “doctors” were replaced with the “long-term care staff” to include the front-line staff that care for residents; the term “patient” was replaced with “resident”).

### 3.2. Phase 2

Cognitive interviews were conducted with a total of 118 LTC family members. The PPS scores for the residents sampled ranged from 80% to 30% (M = 46; SD = 8.89), with almost two-thirds (62%) of the residents having a PPS score of ≤40%. In hospice settings, a PPS score of 40% indicates the need for staff to initiate EOL planning with families (if not previously done) [64]. A PPS score of 30% or less represents a resident requiring EOLC and indicates the need for staff to prepare families for the death and what to expect [65]. Thus, in our research, the PPS scores were beneficial in that they confirmed that the majority of families interviewed were currently experiencing EOL issues with their resident.

The results of the interviews suggested that a few more adaptations were required to make the tool more LTC-relevant and to facilitate understandability.

#### 3.2.1. Revisions to the Questionnaire

*Response Options. *In the initial cognitive interviews (Phase 2), there was no response option for “do not know/no basis to judge.” However, during the interviews, many respondents described being unable to judge their satisfaction with certain care elements because they had not experienced this element of care. For example, many family members said they had no basis to judge their satisfaction with EOL conversations as they had not experienced them. Probing by the interviewer did not elicit a different response. Thus, the “do not know/no basis to judge” response was added to the satisfaction scale.

Families generally commented they “did not know” or had “no basis to judge” if they (i) did not think their family member experienced the medical condition probed by the item, (ii) never saw or met the physician, or (iii) did not have any EOL conversations, either because the staff did not initiate them, the resident was unable (e.g., dementia diagnosis), or the resident was described as unwilling to have these conversations. Illustrative comments include the following: “[resident] is healthy overall, other than limited vision and hearing”; “we never met the doctor”; “[resident] does not want to talk about wishes for future care”; and “[resident] has dementia, so cannot have these discussions.” These comments highlight the unique culture and context of LTC and suggested that this response option was required to account for that uniqueness.

*Wording. *The tense of one item was changed to present because respondents found the past tense confusing (items (14) and (36), Figure 1). Due to families' challenges communicating with residents with cognitive or hearing impairments (common occurrences in LTC), items (22) and (44) were changed from “the discussions with your relative about wishes for future care…” to “you discuss options with the long-term care nurse about your relative's end-of-life care wishes.” In addition, due to the limited nature of physician-family interaction in LTC, the “relationship with doctor” subscale was moved to the second page of the questionnaire.

*Format.* Finally, to avoid navigational confusion on the self-administered tool, the importance and satisfaction scales are presented separately distinguished by textual descriptions.

Thus, the final outcome is a self-administered, 22-item instrument. Respondents rate each item twice (5-point Likert scales): once for importance and once for satisfaction with an option to indicate uncertainty or irrelevance on the satisfaction scale (see Figure 1).

### 3.3. Phase 3

A total of 134 family members returned completed questionnaires (a 75% response rate). The demographic characteristics of respondents are summarized in Table 3.

Most were female, over the age of 55, and a child of the resident. Most visited daily or more than twice a week.

Questionnaire data were analyzed and the results are described below. Descriptive statistics for each item and the importance frequencies can be found in Tables 4 and 5, respectively.

#### 3.3.1. Instrument Properties

*Importance. *The mean importance rating for each item was high (range: 4.13–4.93; Table 4), indicating good content validity. Only half of the items received the full range of endorsements (Table 4). Further indication of content validity is that the proportions of “important” endorsements were high (range: 73–100%), with over one-third (36.4%) of the items not receiving a single “not important” rating (Table 5). Moreover, half of the items (50%) were rated as “extremely important” by 80% or more of respondents (Table 6).

*Satisfaction. *The per-item mean satisfaction ratings ranged from 3.91 to 4.56 (Table 4). The item responses ranged from 1 (not at all) or 2 (not very) to 5 (extremely satisfied) (Table 4). There was some evidence of a ceiling effect as the proportion of 5 (completely satisfied) for any one item ranged from 29.1% to 66.4%. There was little evidence of a floor effect as the proportion of responses of 1 (not at all satisfied) for any one item ranged from 0% to 2.2%.

*Do Not Know/No Basis to Judge. *The proportions of responses of “do not know/no basis to judge” ranged from 0% to 33.6%. Only 5 of the 22 questions had proportions of responses greater than 10%, indicating that “do not know/no basis to judge” is a valid response option.

#### 3.3.2. Internal Consistency

Cronbach's alpha coefficients ranged from .88 to .94 for the overall satisfaction and the five domain scales (Table 7), indicating good-to-excellent results [68].

## 4. Discussion

In the context of the need to develop quality EOLC and measurement tools across diverse EOLC settings and with lack of measures to assess quality of EOLC in LTC, this research was conducted to address the need for a practical tool to measure families' satisfaction during the care experience. The three-phase research process described resulted in an adapted, 22-item, self-administered version of the CANHELP Lite Family Caregiver Questionnaire that is specific to LTC. While preliminary, the results suggest that the adaptations resulted in a validated quality measurement tool.

### 4.1. Revisions to the Questionnaire

This research resulted in several revisions to the CANHELP including some changes to wording and formatting, as well as two rather substantial changes to content.

One content modification was the addition of an item to assess discussions with LTC staff about palliative or comfort care measures. This was added because a literature review revealed that it is an important element of EOLC in LTC: one that was not explicitly probed by the original survey (Phase 1). The results of Phase 3 suggest that this element is indeed very important to family members in the context of LTC as the mean importance rating was very high (M = 4.67, Table 4, item (20)), and 90% of respondents rated it as either “very” or “extremely” important (Table 6). Thus, it is concluded that the addition of that item is justified.

A second content modification was the addition of the “do not know/no basis to judge” response option to the satisfaction scale. It is recognized that providing this response option is not appropriate when it is unlikely to be a genuine response option. In our research, the response option was added intentionally after the initial cognitive interviews suggested that this response was valid in the unique EOLC context of LTC.

During the cognitive interviews, some families expressed that they were not able to validly answer the satisfaction questions as yes/no, despite probing by the interviewer. They genuinely could not answer in many cases as they had not experienced the element being rated. Thus, the “do not know/no basis to judge” option was incorporated as a selection option to minimize a nonattitude reporting [99]. It allows respondents to indicate that they do not know the answer to the question or do not have an opinion on a particular aspect of care. It gives the respondents the ability to be neutral rather than being forced to choose an option.

Given that the proportion of endorsements for the “do not know/no basis to judge” response option ranged from 0 to 34% (Table 4) and only a small proportion of items received endorsements greater than 10%, it is concluded that respondents were not arbitrarily choosing this as an “easy way out.” Rather, this was an honest response. It is therefore concluded that the “do not know/no basis to judge” response option is meaningful in the LTC context, and adding this response option is justified.

### 4.2. Reliability and Validity of the Revised Instrument

In terms of reliability, the revised tool has good internal consistency. Cronbach's alpha coefficient for each of the scales was well above the ≥.80 criterion. Thus, it is concluded that the overall satisfaction scales as well as the five subscales in the revised questionnaire are internally consistent.

The instrument can also be said to have good content and face validity. The revised instrument is based on the original CANHELP which had good content and face validity because the items were generated based on a comprehensive literature review, expert focus groups, and interviews with patients and families. The adaptations made through this research were done to enhance the content and face validity in the unique LTC context. Revisions were made in response to a LTC specific literature review and thorough feedback from family members who were the Powers of Attorney for Personal Care. Because of these revisions, the instrument can be said to adequately cover domains that are relevant to EOLC in LTC and to have language and response options make sense in the LTC context.

The observed importance ratings provide further evidence of content validity and highlight the uniqueness of quality EOLC in LTC. Most family members considered every item to be a “very” or “extremely” important element of good quality care (Table 5), suggesting that the questionnaire is tapping into important elements of EOLC in LTC (i.e., has good content validity). When examining the items ranked by their importance (Table 6), it is interesting to note the trend that elements related to day-to-day care issues (items (1), (4), (6), and (7)) receive higher relative importance ratings than items related to caregiver (item (2)) or EOLC (items (19), (20), and (22)) issues. Those ratings highlight the unique context of EOLC in LTC and differ from families' perspectives in other EOLC settings, where issues related to EOL (e.g., confidence in physician and life sustaining technologies) are of greatest importance [5, 74]. LTC is the residents' home for months or years; residents are thus dying at home as opposed to receiving care in a specialized hospice setting.

### 4.3. Utility and Relevance of Instrument

The population in LTC is different from other EOLC settings in the sense that not all residents are imminently dying. Nevertheless, introducing CANHELP as an EOLC measure at an organizational level is relevant. While only 20% of LTC residents die each year, there are many more residents who are medically unstable and sick enough to die at any time. Gradual decline in their health, punctuated by acute exacerbations of chronic disease, makes prognosis difficult. Best practices for a population with progressive chronic disease, dementia, and frailty are that EOLC should be introduced based on residents' care need rather than prognosis [10, 25, 49, 100]. The chronic disease profile of the LTC population has been described (Table 1). In this research, 60% of residents had PPS scores of 60% or less at the time the family survey was completed. This score indicates that these residents were transitioning (40–60%) or already requiring EOLC (<30%). Given these PPS scores, LTC staff should be explicitly having EOLC conversations with their families for preparation and planning.

A post hoc analysis examined the association of PPS Scores with the “do not know/no basis to judge” response on survey item (42) which asked about family satisfaction with their palliative/comfort care discussions. The results showed no association: *X*^2^ (1, *N* = 124) = .000; *p* = .986. This indicates that families of residents with low PPS scores (e.g., 30% which is bed bound, extensive disease, and total care) were no more likely to have experienced EOL conversations compared to families of residents with higher PPS scores. Regardless of the PPS status of the resident, staff appear not to be having EOL conversations with many family members. This indicates the need for quality improvement because early discussion of goals of care is a best practice in providing EOLC to elderly people with serious illness [91, 92]. The overall QPC-LTC research identified that most LTC staff were not comfortable having advance care planning and EOL conversations with residents and families [27, 28, 30].

The CANHELP Family Caregiver Satisfaction Questionnaire is available in English and French. It has already been adapted for use with family caregivers in specialized care settings including step-down units, chronic respiratory wards, pediatrics, and intensive care. The intensive care version has been translated into many languages: Portuguese, Chinese, German, Hebrew, Greek, Italian, Norwegian, and Swedish. All CANHELP instruments are available and can be downloaded from the CARENET website [57]. Therefore, adapting CANHELP Family Caregiver Satisfaction Questionnaire for LTC contributes to an international research agenda on improving EOLC across settings of care by extending measurement into LTC. The researchers are collaborating with the developers of the CANHELP to further the research using the CANHELP instruments in LTC.

### 4.4. Potential Applications

Like other versions of the CANHELP, one application of the revised questionnaire is to measure satisfaction as an outcome of quality EOLC. LTC homes currently lack a validated instrument to provide consistent outcome measures of EOLC provided at the organizational level. The 22 satisfaction items can be summarized into an overall satisfaction with EOLC in LTC score, and the items in the subscales can be summarized to indicate satisfaction in five subdomains: characteristics of LTC staff, illness management, communication and decision-making, relationship with doctors, and your involvement. In the future, LTC homes could administer this survey to family members annually to measure overall organizational changes in satisfaction scores.

Another application of this questionnaire is in quality improvement. Specifically, the importance scale ratings can be juxtaposed to satisfaction ratings to inform quality improvement strategies at both the individual patient level and overall organization level [57, 101]. For example, items for which there is a large gap between the importance and satisfaction ratings suggest opportunities for quality improvement [57, 101]. Thus, we think that this revised questionnaire can be incorporated into quality improvement programs in LTC homes.

A third application of this instrument is in the analysis of the proportion of per-item “do not know/no basis to judge” responses. Organizational change efforts could be guided by increasing satisfaction scores while reducing “do not know/no basis to judge” responses. For example, in this sample, almost one-third of respondents endorsed this option for item (22), that is, staff members discuss residents EOLC wishes with family members (see Table 4). Knowing residents' wishes is important to guide individualized care planning and decision-making about the location of death. Thus, questionnaire results that indicated high endorsement of the “do not know/no Basis to judge” option may be useful for planning staff education and developing new organizational protocols. For example, our research results suggest that staff may benefit by education on having EOL conversations and the organization may need more explicit protocols to guide implementing EOL conversations [10, 12, 27, 102].

In this research, we surveyed* all *families in LTC with the CANHELP. If desired, the survey could be given only for those families where the resident is transitioning (or has transitioned) to need EOLC. This would mean that an assessment of each resident would precede survey administration and that the survey would only be sent to “targeted” families. The PPS [64, 65] is a simple to use tool that can be used by staff in identifying which residents need or are transitioning to EOLC. LTC homes may also employ other clinical triggers or prognostication tools to identify residents who require EOLC and then survey only these family caregivers.

There are two other potential applications of this instrument which were beyond the scope and purpose of this study. This research only used the CANHELP at the organizational level and did not explore use of the instrument for individual resident care planning. For example, questionnaire results could potentially guide staff in their EOLC discussions during palliative care conferences. This has been done using other versions of the CANHELP and warrants future consideration in LTC.

CANHELP developers have recommended that, for a straightforward quality of care measure, only the satisfaction items are required [57]. Surveying families only for their satisfaction with care would substantially reduce the length of the instrument. Families could also complete the satisfaction items as an online survey. These applications merit further study.

### 4.5. Limitations and Future Research

This research was done as a substudy of the larger QPC-LTC research project, and further psychometric research is needed to fully evaluate this revised tool. Due to the pilot nature of the research (two LTC homes) and the small sample sizes, we were not able to assess the factor structure of the revised instrument or its sensitivity to change; further research in these areas is warranted. With the small sample size, this study lacked the statistical power to assess for overlap of the survey items. However, future research could examine whether there is redundancy among the items or whether there are some domains that are more important than others. Reducing any redundancy would also shorten the questionnaire.

The instrument has 22 items that must be completed for both importance and satisfaction and the length may place a burden on respondents and lower the response rate. However, it is noteworthy that, in the 118 face-to-face interviews conducted for the cognitive interviews, no family member complained about the length of the instrument. In fact, families were very motivated to talk about this topic during the cognitive interviews. The mail-out survey resulted in a response rate of 75%, which suggests that the length of the instrument was not a major concern.

Due to the practical and ethical challenges of interviewing residents noted in the introduction of this paper, we were not able to assess residents' perceptions of quality of care. In the suite of available CANHELP instruments, there is another instrument to measure patient's satisfaction with EOLC which could potentially be adapted for use in LTC. Future research should then correlate residents' satisfaction with families' satisfaction as a measure of criterion-related validity. Similarly, correlations with other instruments were beyond the scope of this research but should be examined in future research to assess the criterion-related validity of this new instrument.

It would also be interesting to assess the tool qualitatively with LTC staff to explore their perceptions on whether they think the items reflect best practice in LTC. In addition, this research was conducted in four LTC homes in Ontario, Canada. Further research is needed to assess the transferability of the resultant questionnaire to other provinces/countries with different LTC structures and policies. Despite these limitations and given the lack of validated instruments available which pertain specifically to LTC, we believe that there is a role for this revised CANHELP in LTC.

Further application and evaluation of the instrument are encouraged. This revised instrument will be mounted on the Canadian Researchers at the End of Life Network (CARENET) website as part of the family of CANHELP instruments [57]. Researchers are encouraged to apply and evaluate this tool and report back on their experience with the instrument. The tool is currently being used in British Columbia (Canada) as part of the Initiative for a Palliative Approach in Nursing: Evidence & Leadership (iPANEL) program of research (http://www.ipanel.ca/); thus, further application and testing of the instrument outside of Ontario (Canada) are currently under way.

## 5. Conclusion

This paper introduces a new tool that can be used to assess families' perceptions of quality and satisfaction with EOLC in LTC. The research provides a new, self-administered version of the CANHELP Lite Individualized Caregiver Questionnaire that is directly applicable to measuring family members' perceptions of EOLC in LTC. While further validation research with this revised instrument is warranted, we conclude that the instrument is internally consistent with good content and face validity. It fills a gap in quality improvement tools to support developing quality PC in LTC.

LTC homes have become a major location of death in Canada and elsewhere. Efforts to implement the palliative approach to care in LTC are underway as their resident population is now very old with chronic illness, frailty, and dementia. Researchers have developed resources to support LTC homes providing palliative and EOLC, including tools and innovations for education, clinical practice, and policy. However, satisfaction measures to measure quality improvement were lacking. This new instrument addresses this gap and may be useful to researchers, practitioners, and administrators for measuring family perceptions of and satisfaction with EOLC in LTC settings.

## Figures and Tables

**Table 1:** Selected characteristics of residents in LTC facilities in Ontario, Canada (2012-2013).

Resident characteristics	
Average age	83 years
Younger than 65 (%)	6.6%
85 and older (%)	53%
Female (%)	68.0%
Total dependence in activities of daily living (%)	12%
Severe cognitive impairment	28.6%
Some indication of health instability (%)	57.2%

Disease diagnosis	

*Endocrine*/*metabolic*/*nutritional diseases*	
Diabetes mellitus	26.6%
Hyperthyroidism	1.1%
Hypothyroidism	18.5%
*Heart*/*circulation diseases*	
Arteriosclerotic heart disease	14.3%
Cardiac dysrhythmia	7.7%
Congestive heart failure	12.8%
Deep vein thrombosis	1.5%
Hypertension	61.3%
Hypotension	1.4%
Other cardiovascular diseases	15.0%
Peripheral vascular disease	6.1%
*Musculoskeletal diseases*	
Arthritis	59.9%
Hip fracture	42.4%
Missing limb	6.5%
Osteoporosis	1.1%
Pathological bone fracture	29.8%
*Neurological diseases*	
Dementia	61.1%
Amyotrophic lateral sclerosis (ALS)	0.2%
Aphasia	8.1%
Cerebral palsy	0.6%
Cerebrovascular accident (stroke)	22.1%
Hemiplegia/hemiparesis	6.3%
Huntington's chorea	0.3%
Multiple sclerosis	1.3%
Paraplegia	0.5%
Parkinson's disease	7.0%
Quadriplegia	0.3%
Seizure disorder	5.5%
Transient ischemic attack (TIA)	5.5%
Traumatic brain injury	1.0%
*Pulmonary diseases*	
Asthma	4.1%
Emphysema/COPD	15.8%
*Other diseases*	
Allergies	27.4%
Anemia	16.5%
Cancer	8.4%
Gastrointestinal disease	20.6%
Liver disease	1.1%
Renal failure	10.2%

*Source*: Canadian Institute for Health Information [9].

**Table 2:** Questions (and their rationales) used in the cognitive interviews.

Interview question	Rationale
Were these questions easy or difficult to answer?	To determine comprehension and overall ability to recall
How did you find the order of the questions presented in the survey?	To explore the structure of the questionnaire
What was your perception on answering questions regarding your “Relationship with Doctor(s)”?	To explore whether physician interaction was a concern in LTC setting
How did you decide on your importance rating? And how did you decide on your satisfaction rating?	To explore internal and external estimation or judgement cues
Were these decisions easy or difficult to make?	To assess comprehension and burden of the questions
How sure were you of the answers?	Confidence probe
Were there any terms or concepts that were unclear?	To test interpretation of specific terms

**Table 3:** Demographic characteristics of the survey respondents (*N* = 134).

Characteristic	(%)^a^
Gender	
Male	26.9
Female	70.1
Age	
<55	15.7
55–64	42.5
65–74	27.6
75–84	10.4
85–94	1.5
Education	
Elementary	1.5
Some high school	7.5
High school graduate	20.1
Some college	12.7
College diploma	15.7
Some university	10.4
University degree	21.6
Postgraduate	7.5
Relation to resident	
Spouse	15.7
Parent	4.5
Sibling	2.2
Child	64.2
Other	10.4
Ethnicity	
Caucasian	94.8
Other	3.0
Visit frequency^b^	
Daily	23.1
4–6 times per week	5.2
2-3 times per week	19.4
Weekly	42.5
Biweekly	4.5
Monthly	3.0

^a^Totals do not equal 100 due to missing data. ^b^Answer to question “how often do you visit the resident?”

**Table 4:** Mean, standard deviations, response range, and proportion of do not know/no basis to judge endorsement for each item on the revised questionnaire.

Item^a^	Importance scale			Satisfaction scale			% DK/NBJ^c^
	Mean	SD	Range^b^	Mean	SD	Range^b^	
(1) Staff are compassionate/supportive of resident	4.93	.28	3–5	4.41	.74	1–5	—
(2) Staff are compassionate/supportive of family member	4.13	.91	1–5	4.40	.72	2–5	1.5
(3) Tests/treatments are given in the LTC home	4.69	.52	3–5	4.47	.77	1–5	2.2
(4) Physical symptoms adequately assessed/managed	4.88	.35	3–5	4.40	.85	1–5	4.5
(5) Emotional problems adequately assessed/controlled	4.77	.49	3–5	4.26	.93	1–5	5.2
(6) Help with personal care when needed	4.90	.33	3–5	4.41	.79	2–5	—
(7) Care received when family not there	4.87	.38	3–5	4.37	.77	1–5	3.7
(8) Health care workers work as a team	4.81	.40	4-5	4.44	.72	1–5	4.5
(9) Management of financial costs	4.54	.68	2–5	4.41	.86	1–5	.7
(10) Environment is calm and restful	4.60	.60	3–5	4.35	.81	1–5	—
(11) The care/treatment is consistent with wishes	4.61	.67	1–5	4.34	.80	1–5	6.0
(12) Staff communicate to you straightforwardly/honestly	4.80	.55	1–5	4.55	.78	1–5	1.5
(13) Receive consistent information about resident's condition	4.74	.61	1–5	4.35	.88	1–5	2.2
(14) The LTC staff listen to what you say	4.77	.51	2–5	4.40	.83	2–5	1.5
(15) Discuss options about hospital transfer with the staff	4.64	.75	1–5	4.46	.87	1–5	17.9
(16) LTC home doctor takes a personal interest in relative	4.62	.70	1–5	4.07	.96	1–5	9.0
(17) LTC home doctor available when needed	4.57	.76	1–5	3.91	1.04	1–5	15.7
(18) Trust and confidence in the doctor	4.77	.52	2–5	4.10	1.02	1–5	6.7
(19) Discuss options with staff about life sustaining technologies	4.55	.87	1–5	4.45	.88	1–5	21.6
(20) You discuss palliative/comfort care measures with staff	4.67	.66	1–5	4.51	.68	2–5	33.6
(21) Your role in decision-making about medical care LTC home	4.74	.63	1–5	4.55	.71	2–5	7.5
(22) Discuss options with staff about relative's EOLC wishes	4.59	.83	1–5	4.56	.67	2–5	32.1

^a^Items are abbreviated; full wording can be seen in Figure 1. ^b^Item range potentials were 1–5 for the importance scale and 1–5 for satisfaction scale with an additional option to select “do not know/no basis to judge.” Values in the table are the observed ranges for each item. ^c^DK/NBJ: do not know/no basis to judge response option.

**Table 5:** Family members' importance ratings of elements related to quality end-of-life care in long-term care home.

Item^a^	Frequency (%)		
	Not important^b^	Somewhat important	Important^c^
*Characteristics of LTC staff*			
(1) Staff are compassionate/supportive of resident	—	.7	99.2
(2) Staff are compassionate/supportive of family member	2.9	23.9	73.2
*Illness management*			
(3) Tests/treatments are given in the LTC home	—	2.2	97.8
(4) Physical symptoms adequately assessed/managed	—	.7	98.5
(5) Emotional problems adequately assessed/controlled	—	3.0	96.3
(6) Help with personal care when needed	—	.7	99.3
(7) Care received when family not there	—	1.5	98.5
(8) Health care workers work as a team	—	—	100
(9) Management of financial costs	1.5	6.0	89.6
(10) Environment is calm and restful	—	6.0	94.0
(11) The care/treatment is consistent with wishes	1.4	3.7	94.0
*Communication and decision-making*			
(12) Staff communicate to you straightforwardly/honestly	.7	2.2	97.0
(13) Receive consistent information about resident's condition	.7	4.5	94.8
(14) The LTC staff listen to what you say	.7	1.5	97.0
(15) Discuss options about hospital transfer with the staff	2.2	4.5	90.3
*Relationship with doctors*			
(16) LTC home doctor takes a personal interest in relative	2.2	3.7	93.3
(17) LTC home doctor available when needed	2.2	4.5	90.3
(18) Trust and confidence in the doctor	.7	2.2	95.6
*Your involvement*			
(19) Discuss options with staff about life sustaining technologies	3.7	4.5	90.3
(20) You discuss palliative/comfort care measures with staff	1.4	3.0	90.2
(21) Your role in decision-making about medical care LTC home	1.4	3.0	94.8
(22) Discuss options with staff about relative's EOLC wishes	3.4	5.2	87.3

^a^Items are abbreviated; full wording can be seen in Figure 1. ^b^Combined percentage of “not at all” and “not very” important ratings. ^c^Combined percentage of “very” and “extremely” important ratings.

*Note*. Not all frequencies sum to 100 because of some missing data/no response.

**Table 6:** Family members' ranked importance ratings of elements related to quality end-of-life care in long-term care home.

Rank^a^	Care element (item)^b^	Rating, % respondents (*n* = 134)	
		Extremely	Very
1	Staff are compassionate/supportive of resident (1)	94	5.2
2	Help with personal care when needed (6)	90.3	9
3	Care received when family not there (7)	88.8	9.7
4	Physical symptoms adequately assessed/managed (4)	88.1	10.4
5	Staff communicate to you straightforwardly/honestly (12)	84.3	12.7
6	Receive consistent information about resident's condition (13)	80.6	14.2
6	Health care workers work as a team (8)	80.6	19.4
7	Trust and confidence in the doctor (18)	79.9	15.7
7	Your role in decision-making about medical care LTC home (21)	79.9	14.9
7	The LTC staff listen to what you say (14)	79.7	17.9
7	Emotional problems adequately assessed/controlled (5)	79.7	17.2
8	Discuss options about hospital transfer with the staff (15)	72.4	17.9
9	Tests/treatments are given in the LTC home (3)	70.9	26.9
9	LTC home doctor takes a personal interest in relative (16)	70.9	22.4
9	You discuss palliative/comfort care measures with staff (20)	70.1	20.1
9	Discuss options with staff about relative's EOLC wishes (22)	70.1	17.5
10	Discuss options with staff about life sustaining technologies (19)	69.4	20.9
11	The care/treatment is consistent with wishes (11)	67.9	26.1
12	Environment is calm and restful (10)	66.4	27.6
13	LTC home doctor available when needed (17)	65.7	24.6
14	Management of financial costs (9)	61.2	28.4
15	Staff are compassionate/supportive of family member (2)	43.3	29.9

^a^Ranked by the proportion of respondents who rated the element as “extremely important.” ^b^Number corresponds to the questionnaire item in Figure 1. Item wording in Table 4 is abbreviated. See Figure 1 for full wording of items.

**Table 7:** Cronbach's alpha for the adapted CANHELP Lite-LTC Family Questionnaire.

Questionnaire	Number of items	Cronbach's alpha coefficient	95% confidence interval
Overall satisfaction	22	.94	.92–.96
Characteristics of LTC staff	2	.93	.90–.95
Illness management	9	.90	.86–.91
Communication and decision-making	4	.92	.89–.94
Relationships with doctors	3	.92	.89–.94
Your involvement	4	.88	.83–.92

## References

[1] Carstairs S., Beaudoin G. A. (2000). *Quality End of Lifecare: The Right of Every Canadian*.

[2] Giovanni L. A. (2012). End-of-life care in the United States: current reality and future promise—a policy review. *Nursing Economics*.

[3] NHS England Actions for End of Life Care: 2014-16. http://www.england.nhs.uk/wp-content/uploads/2014/11/actions-eolc.pdf.

[4] Dy S., Shugarman L., Lorenz K., Mularski R., Lynn J. (2008). A systematic review of satisfaction with care at the end of life. *Journal of the American Geriatrics Society*.

[5] Heyland D. K., Cook D. J., Rocker G. M. (2010). Defining priorities for improving end-of-life care in Canada. *Canadian Medical Association Journal*.

[6] Lo B. (1995). Improving care near the end of life. Why is it so hard?. *Journal of the American Medical Association (JAMA)*.

[7] Mularski R., Dy S., Shugarman L. (2007). Systematic review of measures of end-of-life care and its outcomes. *Health Services Research*.

[8] Stewart A. L., Teno J., Patrick D. L., Lynn J. (1999). The concept of quality of life of dying persons in the context of health care. *Journal of Pain and Symptom Management*.

[9] Canadian Institute for Health Information (2012) Continuing care reporting system, Quick Stats Tables, Profile of Residents in Continuing Care Facilities 2012-2013. https://www.cihi.ca/en.

[10] Brink P., Kelley M. L. (2015). Death in long-term care: a brief report examining factors associated with death within 31 days of assessment. *Palliative Care: Research and Treatment*.

[11] Statistics Canada (2011). Residential care facilities, 2009-2010. *Catalogue no. 83-237-X. Ministry of Industry, Health Statistics Division*.

[12] Marcella J., Kelley M. L. (2015). Death is part of the job in long-term care homes: supporting direct care staff with their grief and bereavement. *SAGE Open*.

[13] Munn J. C., Dobbs D., Meier A., Williams C. S., Biola H., Zimmerman S. (2008). The end-of-life experience in long-term care: five themes identified from focus groups with residents, family members, and staff. *The Gerontologist*.

[14] McAuley W. J., Buchanan R. J., Travis S. S., Wang S., Kim M. (2006). Recent trends in advance directives at nursing home admission and one year after admission. *The Gerontologist*.

[15] Blackford J., Strickland E., Morris B. (2007). Advance care planning in residential aged care facilities. *Contemporary Nurse*.

[16] Mallery L. H., Moorhouse P. (2010). Respecting frailty. *Journal of Medical Ethics*.

[17] Cantor M. C., Pearlman R. A. (2003). Advance care planning in long-term care facilities. *Journal of the American Medical Directors Association*.

[18] Canadian Hospice Palliative Care Association [CHPCA] & Quality End-of-Life Care Coalition of Canada (2015) The Way Forward National Framework: A Roadmap for an Integrated Palliative. http://www.hpcintegration.ca.

[19] World Health Organization [WHO] 2004 Better Palliative Care for Older People. http://www.euro.who.int/__data/assets/pdf_file/0009/98235/E82933.pdf.

[20] World Health Organization (2015) WHO Definition of Palliative Care. http://www.who.int/cancer/palliative/definition/en/.

[21] Canadian Hospice Palliative Care Association [CHPCA] 2013 Model to Guide Hospice Palliative Care: Based on National Principles and Norms of Practice. http://www.chpca.net/media//norms-of-practice-eng-web.pdf.

[22] Quality Palliative Care in Long-Term Care Alliance (2015) Philosophy of Palliative Care, Providing Care for Life: Resident Transitions in Long Term Care. http://www.palliativealliance.ca/project-results#fig2.

[23] Rocker G., Downar J., Morrison R. S. (2016). Palliative care for chronic illness: driving change. *Canadian Medical Association Journal*.

[24] Sawatzky R., Porterfield P., Lee J. (2016). Conceptual foundations of a palliative approach: a knowledge synthesis. *BMC Palliative Care*.

[25] Quality Palliative Care in Long Term Care: A Community-University Research Alliance. QPC –LTC Project. http://www.palliativealliance.ca.

[26] Kelley M. L., McKee M., Hockley J., Froggatt K., Heimerl K. (2013). Community capacity development in participatory action research. *Participatory Research in Palliative Care: Actions and Reflections*.

[27] Ramsbottom K., Kelley M. L. (2014). Developing strategies to improve advance care planning in long term care homes: giving voice to residents and their family members. *International Journal of Palliative Care*.

[28] Kortes-Miller K., Jones-Bonofiglio K., Hendrickson S., Kelley M. L. (2016). Dying with carolyn: using simulation to improve communication skills of unregulated care providers working in long-term care. *Journal of Applied Gerontology*.

[29] Vis J., Ramsbottom K., Marcella J. (2016). Developing and implementing peer-led intervention to support staff in long-term care homes manage grief. *Sage Open*.

[30] Sims-Gould J., Wiersma E., Kelley M. L. (2010). Perspectives on end-of-life care in long-term care homes: implications for whole person and palliative care. *Journal of Palliative Care*.

[31] Wickson-Griffiths A., Kaasalainen S., Brazil K. (2015). Comfort care rounds: a staff capacity-building initiative in long-term care homes. *Journal of Gerontological Nursing*.

[32] Brazil K., Brink P., Kaasalainen S., Kelley M. L., McAiney C. (2012). Knowledge and perceived competence among nurses caring for the dying in long-term care homes. *International Journal of Palliative Nursing*.

[33] Wickson-Griffiths A., Kaasalainen S., Ploeg J., McAiney C. (2014). A review of advance care planning programs in long-term care homes: are they dementia friendly?. *Nursing Research & Practice*.

[34] Landau L., Brazil K., Kaasalainen S., Crawshaw D. (2013). Training and sustaining: a model for volunteer spiritual care visitors in long-term care. *Journal of Religion, Spirituality and Aging*.

[35] Larocque N., Schotsman C., Kaasalainen S., Crawshaw D., McAiney C., Brazil E. (2014). Using a book chat to improve attitudes and perceptions of long-term care staff about dementia. *Journal of Gerontological Nursing*.

[36] Kaasalainen S., Brazil K., Kelley M. L. (2014). Building capacity in palliative care for personal support workers in long-term care through experiential learning. *International Journal of Older People Nursing*.

[37] Levin J. R., Wenger N. S., Ouslander J. G. (1999). Life-sustaining treatment decisions for nursing home residents: who discusses, who decides and what is decided?. *Journal of the American Geriatrics Society*.

[38] Cohen R. S., Sawatsky R., Russell L. B., Shahidi J., Heyland D. K. (2017). Measuring the quality of life of people at the end of life: the mcgill quality of life questionnaire-revised. *Palliative Medicine*.

[39] Robin Cohen S., Mount B. M., Strobel M. G., Bui F. (1995). The McGill quality of life questionnaire: a measure of quality of life appropriate for people with advanced disease. A preliminary study of validity and acceptability. *Palliative Medicine*.

[40] Kane R. A., Ling K. C., Bershadsky B. (2003). Quality of life measures for nursing home residents. *Journals of Gerontology Series A*.

[41] Kane R. A. (2001). Long-term care and a good quality of life: bringing them closer together. *The Gerontologist*.

[42] Subasi F., Hayran O. (2005). Evaluation of life satisfaction index of the elderly people living in nursing homes. *Archives of Gerontology and Geriatrics*.

[43] Tseng S., Wang R. (2001). Quality of life and related factors among elderly nursing home residents in Southern Taiwan. *Public Health Nursing*.

[44] Zimmerman S., Sloane P. D., Williams C. S. (2005). Dementia care and quality of life in assisted living and nursing homes. *The Gerontologist*.

[45] Vohra J. U., Brazil K., Hanna S., Abelson J. (2004). Family perceptions of end-of-life care in long-term care facilities. *Journal of Palliative Care*.

[46] Wetle T., Shield R., Teno J., Miller S. C., Welch L. (2005). Family perspectives on end-of-life care experiences in nursing homes. *The Gerontologist*.

[47] Wilson S. A., Daley B. J. (1999). Family perspectives on dying in long-term care settings. *Journal of Gerontological Nursing*.

[48] Teno J. M., Clarridge B., Casey V., Edgman-Levitan S., Fowler J. (2001). Validation of toolkit after-death bereaved family member interview. *Journal of Pain and Symptom Management*.

[49] Teno J. M., Casey V. A., Welch L. C., Edgman-Levitan S. (2001). Patient-focused, family-centered end-of-life medical care: views of the guidelines and bereaved family members. *Journal of Pain and Symptom Management*.

[50] Teno J. M., Clarridge B. R., Casey V. (2004). Family perspectives on end-of-life care at the last place of care. *The Journal of the American Medical Association*.

[51] Vohra J. U., Brazil K., Szala-Meneok K. (2006). The last word: family members' descriptions of end-of-life care in long-term care facilities. *Journal of Palliative Care*.

[52] Oliver D. P., Porock D., Zweig S. (2005). End-of-life care in U.S. nursing homes: a review of the evidence. *Journal of the American Medical Directors Association*.

[53] Thompson G. N., McClement S. E., Menec V. H., Chochinov H. M. (2012). Understanding bereaved family members dissatisfaction with end-of-life care in nursing homes. *Journal of Gerontological Nursing*.

[54] Heyland D. K., Cook D. J., Rocker G. M. (2010). The development and validation of a novel questionnaire to measure patient and family satisfaction with end-of-life care: The Canadian Health Care Evaluation Project (CANHELP) questionnaire. *Palliative Medicine*.

[55] Heyland D. K., Jiang X., Day A. G., Cohen S. R. (2013). The development and validation of a shorter version of The Canadian Health Care Evaluation Project Questionnaire (CANHELP Lite): a novel tool to measure patient and family satisfaction with end-of-life care. *Journal of Pain and Symptom Management*.

[56] Heyland D. K., Cook D. J., Rocker G. M. (2010). Defining priorities for improving end-of-life care in canada. *Canadian Medical Association Journal*.

[57] http://thecarenet.ca/resource-center/canhelp.

[58] Brazil K., McAiney C., Caron-O'brien M., Kelley M. L., O'Krafka P., Sturdy-Smith C. (2004). Quality end-of-life care in long-term care facilities: service providers' perspective. *Journal of Palliative Care*.

[59] Kaasalainen S., Coker E., Dolovich L. (2007). Pain management decision making among long-term care physicians and nurses. *Western Journal of Nursing Research*.

[60] Streiner D. L. (1993). A checklist for evaluating the usefulness of rating scales. *Canadian Journal of Psychiatry*.

[61] Attorney General, Government of Ontario, Canada. The Substitute Decisions Act. https://www.ontario.ca/laws/statute/92s30

[62] Willis G. B. (2005). *Cognitive Interviewing: A Tool for Improving Questionnaire Design*.

[63] Willis G. Cognitive Interviewing: A “How To” Guide. http://citeseerx.ist.psu.edu/viewdoc/download;jsessionid=71CC295F5AF0F2D2C4B4804BF5CE5C9C?doi=10.1.1.469.6024&rep=rep1&type=pdf.

[64] Victoria Society Hospice (2001) The palliative performance scale version 2 (PPSv2). http://www.npcrc.org/files/news/palliative_performance_scale_PPSv2.pdf.

[65] Anderson F., Downing G. M. (1996). Palliative performance scale (PPS): a new tool. *Journal of Palliative Care*.

[66] Cairns M., Thompson M., Wainwritght W. (2016). *Transitions in Dying Bereavement: A Psychosocial Guide for Hospice Palliative Care*.

[67] Head B., Ritchie C. S., Smoot T. M. (2005). Prognostication in hospice care: can the palliative performance scale help?. *Journal of Palliative Medicine*.

[68] Tabachnick B. G., Fidell L. S. (2006). *Using Multivariate Statistics*.

[69] Engel S. E., Kiely D. K., Mitchell S. L. (2006). Satisfaction with end-of-life care for nursing home residents with advanced dementia. *Journal of the American Geriatrics Society*.

[70] Forbes S. (2001). This is heaven's waiting room: end of life in one nursing home. *Journal of Gerontological Nursing*.

[71] Sloane P. D., Zimmerman S., Hanson L., Mitchell C. M., Riedel-Leo C., Custis-Buie V. (2003). End-of-life care in assisted living and related residential care settings: comparison with nursing homes. *Journal of the American Geriatrics Society*.

[72] Rogers A., Karlsen S., Addington-Hall J. (2000). Dissatisfaction with care in the last year of life. *Journal of Advanced Nursing*.

[73] Cherlin E., Schulman-Green D., McCorkle R., Johnson-Hurzeler R., Bradley E. (2004). Family perceptions of clinicians' outstanding practices in end-of-life care. *Journal of Palliative Care*.

[74] Heyland D. K., Dodek P., Rocker G. (2006). What matters most in end-of-life care: perceptions of seriously ill patients and their family members. *Canadian Medical Association Journal*.

[75] Gladstone J., Wexler E. (2002). Exploring the relationships between families and staff caring for residents in long-term care facilities: family members' perspectives. *Canadian Journal on Aging*.

[76] Stewart M. A. (1995). Effective physician-patient communication and health outcomes: a review. *Canadian Medical Association Journal*.

[77] Cramer G. W., Galer B. S., Mendelson M. A., Thompson G. D. (2000). A drug use evaluation of selected opioid and nonopioid analgesics in the nursing facility setting. *Journal of the American Geriatrics Society*.

[78] Ferrell B. A. (2004). The management of pain in long-term care. *Clinical Journal of Pain*.

[79] Kayser-Jones J. (2002). The experience of dying: an ethnographic nursing home study. *The Gerontologist*.

[80] Reynolds K., Henderson M., Schulman A., Hanson L. C. (2002). Needs of the dying in nursing homes. *Journal of Palliative Medicine*.

[81] Steele L. L., Mills B., Long M. R., Hagopian G. A. (2002). Patient and caregiver satisfaction with end-of-life care: does high satisfaction mean high quality of care?. *American Journal of Hospice & Palliative Care*.

[82] Singer P. A., Martin D. K., Kelner M. (1999). Quality end-of-life care: patients' perspectives. *Journal of the American Medical Association*.

[83] Ersek M., Wilson S. A. (2003). The challenges and opportunities in providing end-of-life care in nursing homes. *Journal of Palliative Medicine*.

[84] Hanson L. C., Danis M., Garrett J. (1997). What is wrong with end-of-life care? Opinions of bereaved family members. *Journal of the American Geriatrics Society*.

[85] Shiozaki M., Morita T., Hirai K., Sakaguchi Y., Tsuneto S., Shima Y. (2005). Why are bereaved family members dissatisfied with specialised inpatient palliative care service? A nationwide qualitative study. *Palliative Medicine*.

[86] Emanuel E. J., Fairclough D. L., Wolfe P., Emanuel L. L. (2004). Talking with terminally ill patients and their caregivers about death, dying, and bereavement: Is it stressful? is it helpful?. *Archives of Internal Medicine*.

[87] Keegan O., McGee H., Hogan M., Kunin H., O'Brien S., O'Siorain L. (2001). Relatives' views of health care in the last year of life. *International Journal of Palliative Nursing*.

[88] Gruneir A. (2013). Avoidable, emergency department transfers from long-term care homes: a brief review. *Healthcare Quarterly*.

[89] Evans W. G., Cutson T. M., Steinhouser K. E., Tulsky J. A. (2006). Is there no place like home? Caregivers recall reasons for and experience upon transfer from home hospice to inpatient facilities. *Journal of Palliative Medicine*.

[90] Curtis J. R., Patrick D. L., Engelberg R. A., Norris K., Asp C., Byock I. (2002). A measure of the quality of dying and death: initial validation using after-death interviews with family members. *Journal of Pain and Symptom Management*.

[91] You J. L., Fowler R. A., Heyland D. K. (2014). Just ask: discussing goals of care with patients in hospital with serious illness. *Canadian Medical Association Journal*.

[92] Heyland D. K., Barwich D., Pichora D. (2013). Failure to engage hospitalized elderly patients and their families in advance care planning. *JAMA Internal Medicine*.

[93] Moorhouse P., Mallery L. H. (2012). Palliative and therapeutic harmonization (PATH): a new model for decision making in frail older adults. *Journal of the American Geriatrics Society*.

[94] Bostick J. E., Rantz M. J., Flesner M. K., Riggs C. J. (2006). Systematic review of studies of staffing and quality in nursing homes. *Journal of the American Medical Directors Association*.

[95] Miller S. C., Teno J. M., Morgan V. (2004). Hospice and palliative care in nursing homes. *Clinics in Geriatric Medicine*.

[96] Dawson N. J. (1991). Need satisfaction in terminal care settings. *Social Science and Medicine*.

[97] Raudonis B. M., Kyba F. C., Kinsey T. A. (2002). Long-term care nurses' knowledge of end-of-life care. *Geriatric Nursing*.

[98] Rice K. N., Coleman E. A., Fish R., Levy C., Kutner J. S. (2004). Factors influencing models of end-of-life care in nursing homes: results of a survey of nursing home administrators. *Journal of Palliative Medicine*.

[99] Krosnick J. A., Holbrook A. L., Berent M. K. (2002). The impact of ‘no opinion’ response options on data quality. *Public Opinion Quarterly*.

[100] Hockley J., Dewar B., Watson J. (2005). Promoting end-of-life care in nursing homes using an integrated care pathway for the last days of life. *Journal of Research in Nursing*.

[101] Frank C., Touw M., Suurdt J., Jiang X., Wattam P., Heyland D. K. (2012). Optimizing end of life care on medical clinical teaching units using the canhelp questionnaire and a nurse facilitator: a feasibility study. *Canadian Journal of Nursing Research*.

[102] Travis S. S., Bernard M., Dixon S., McAuley W. J., Loving G., McClanahan L. (2002). Obstacles to palliation and end-of-life care in a long-term care facility. *Gerontologist*.

